# Local Neuronal Ensembles That Coreactivate across Regions during Sleep Are Preferentially Stabilized

**DOI:** 10.1523/JNEUROSCI.1125-25.2025

**Published:** 2025-12-11

**Authors:** Hiroyuki Miyawaki, Kenji Mizuseki

**Affiliations:** Department of Physiology, Graduate School of Medicine, Osaka Metropolitan University, Osaka 545-8585, Japan

**Keywords:** amygdalar high-frequency oscillations, cell ensembles, cortical ripples, inter-regional coactivation, neuronal reactivation, sharp-wave ripples

## Abstract

Neuronal ensembles, which represent coordinated activity patterns within individual brain regions, play crucial roles in memory. While local ensembles dynamically change in response to experience, factors influencing the stability of neuronal ensemble compositions remain largely unknown. In this study, we analyzed the dynamics of neuronal ensembles in the prelimbic cortex layer 5 (PL5), basolateral nucleus of the amygdala (BLA), and ventral hippocampus CA1 region (vCA1) during conditioning, extinction, retention of extinction, and interleaving sleep epochs in fear-conditioned male rats. We found that ensemble compositions in the PL5 were more stable than those in the BLA and vCA1. Ensemble reactivation during non-rapid eye movement (NREM) sleep following extinction sessions did not fully explain whether extinction-related ensembles were preserved until the retention-of-extinction sessions. Although the extinction sessions did not affect the number of ensemble pairs that were inter-regionally coactivated during NREM sleep, patterns of inter-regional ensemble coactivation were reorganized after extinction sessions, suggesting that extinction modifies the fine-scale structure of inter-regional networks. Notably, extinction-related ensembles that coactivated with those in other regions during post-extinction NREM sleep were more likely to be preserved until the retention-of-extinction sessions. In post-extinction NREM sleep, the preserved extinction-related ensembles contributing to inter-regional coactivation were activated more frequently during fast network oscillations. These findings suggest that local ensembles contributing to broader inter-regional activity are preferentially stabilized, supporting a systems-level mechanism of memory consolidation.

## Significance Statement

Neuronal ensembles, relatively small populations of simultaneously activated neurons within a brain region, encode specific information. Since ensemble compositions change with experience, factors that stabilize local ensembles are critical for advancing our understanding of memory mechanisms and developing translational applications to treat memory-related disorders. While previous research has primarily focused on local mechanisms underlying ensemble stabilization, the role of inter-regional interactions has largely remained unexplored. Our study revealed that local ensembles that coactivate with ensembles in other brain regions are more likely to be preserved over time. Moreover, preserved ensembles contributing to inter-regional coactivation are reactivated more frequently during fast network oscillations in sleep, suggesting an important role of inter-regional networks activated during fast network oscillations in stabilizing local ensembles.

## Introduction

The brain processes diverse information across multiple regions and integrates the results to support adaptive behavior (Mesulam, 1998; Miller and Cohen, 2001). Animals modify their actions based on experience, implying that experience alters how information is processed within individual regions and/or how information is integrated across regions (Buonomano and Merzenich, 1998). However, how experience-induced changes occur within- and inter-regional networks and how these changes interact remain unclear.

Recent studies have revealed that neuronal ensembles, small populations of coactive neurons within a region, encode specific information (Liu et al., 2012; Tonegawa et al., 2015). Memory-related ensembles or memory engrams (Josselyn and Tonegawa, 2020) exist prior to experience (Dragoi and Tonegawa, 2011; Kim et al., 2014; Mizuseki and Miyawaki, 2017; Miyawaki and Mizuseki, 2022) and are modified through experience (Schoenenberger et al., 2016; Carrillo-Reid et al., 2023). Ensembles active during wakefulness are reactivated during subsequent sleep in various regions, including the hippocampus (Wilson and McNaughton, 1994; Skaggs and McNaughton, 1996; Lee and Wilson, 2002; Dupret et al., 2010), neocortex (Ji and Wilson, 2007; Peyrache et al., 2009; Miyamoto et al., 2016), and amygdala (Girardeau et al., 2017). Such reactivation stabilizes local ensembles (Grosmark and Buzsáki, 2016) with modest reorganizations (Ghandour et al., 2019; Maboudi et al., 2024; Bollmann et al., 2025) and perturbing the reactivation impairs memory consolidation (Girardeau et al., 2009; Gridchyn et al., 2020). These findings suggest that local ensembles are modified through memory consolidation. However, the extent to which local ensembles are modified through memory processes, and mechanisms by which this occurs, remain poorly understood.

Memory-related ensembles emerge concurrently across multiple regions (Roy et al., 2022), and inter-regional coactivations of these ensembles develop after experience (Miyawaki and Mizuseki, 2022; Mizuseki and Miyawaki, 2023). Such coactivations likely reflect inter-regional communication, an important process in systems memory consolidation (Diekelmann and Born, 2010). Indeed, hippocampocortical coactivation during sleep facilitates changes in cortical circuits (Ji and Wilson, 2007; Peyrache et al., 2009; Maingret et al., 2016; Kitamura et al., 2017), and memory-contributing neurons in one region retrogradely modulate ensemble compositions in other regions (Lavi et al., 2023). Furthermore, fast network oscillations, such as hippocampal sharp-wave ripples (SWRs), cortical ripples (cRipples), and amygdala high-frequency oscillations (HFOs), are prominent during sleep, frequently coupled across regions and support memory consolidation (Ponomarenko et al., 2003; Girardeau et al., 2009; Peyrache et al., 2009; Girardeau et al., 2017; Headley et al., 2017; Khodagholy et al., 2017; Miyawaki and Mizuseki, 2022; Mizuseki and Miyawaki, 2023; Kajiya et al., 2025). Together, these observations highlight the importance of coordinated inter-regional activity during fast network oscillations in memory consolidation.

Fear conditioning and extinction provide well-established paradigms for investigating memory acquisition and consolidation. Both processes involve the amygdala, prefrontal cortex, and hippocampus (Tronson et al., 2012; Tovote et al., 2015). Extinction does not erase the original fear memory but instead forms a new memory that suppresses the conditioned response (Bouton, 1993; Quirk and Mueller, 2008; Milad and Quirk, 2012). The extinction memory undergoes consolidation (Santini et al., 2004), but spontaneous recovery of the fear response frequently occurs (Bouton, 1993), indicating dynamic and flexible neural network interactions in extinction learning (Milad and Quirk, 2012). These findings suggest that the dynamics of extinction-related neuronal ensembles are likely to be diverse and that their stability is influenced by inter-regional network interactions. However, despite the translational importance for treating post-traumatic stress disorder, the mechanisms that stabilize extinction-related ensembles remain largely unknown.

To identify factors affecting the stability of extinction-related ensembles, we analyzed previously obtained ∼17 h continuous recordings of unit activity and local field potentials (LFPs) from the prelimbic cortex layer 5 (PL5), basolateral nucleus of the amygdala (BLA), and ventral hippocampus CA1 region (vCA1) of fear-conditioned rats (Miyawaki and Mizuseki, 2022), focusing on the dynamics of ensembles detected during extinction sessions. We found that both local and inter-regional networks changed following extinction sessions. Furthermore, extinction-related local ensembles were more likely to be preserved when they participated in inter-regional coactivation.

## Materials and Methods

### Surgery and data collection

We analyzed previously recorded unit activity and LFPs from the vCA1, PL5, and BLA of male Long–Evans rats (Miyawaki and Mizuseki, 2022). Only male rats were used to exclude any potential effects of estrous cycles on neural activities and animal behaviors. All animal experiments were conducted in accordance with the National Institutes of Health guidelines and approved by the Institutional Animal Care and Use Committee of Osaka City University (approval #15030).

Briefly, 15 rats (aged 9.6–15.0 weeks) were anesthetized with 1–3% isoflurane (in a 50% air/50% oxygen gas mixture). Stainless steel wires for electrocardiography (ECG) and electromyography (EMG) recordings were inserted into the left intercostal and nuchal muscles, respectively. Screws for reference, ground, and electro-olfactography (EOG) recordings were placed on the skull. Tungsten wires for electrical stimulation were inserted into the eyelids (Johansen et al., 2010). Three 64-channel silicon probes (Buzsaki64sp and Buzsaki64spL from Neuronexus or F6–64 from Cambridge Neurotech) targeting the PL5, BLA, and vCA1 were implanted through 1 × 2 mm cranial windows centered at [anterior +2.90 to +3.25 mm, lateral +1.0 to +1.5 mm], [anterior −2.60 to −3.00 mm, lateral +4.60 to +4.80 mm], and [anterior −4.95 to −5.55 mm, lateral +2.80 to +3.00 mm] relative to the bregma, respectively.

After 3–15 d of recovery, signals from all implanted probes, as well as EMG, ECG, and EOG, were continuously recorded for ∼17 h using a C3100 256-channel acquisition board (Intan) or a 512-channel acquisition board (Open Ephys). During data collection, the rats underwent a fear-conditioning experiment consisting of five behavioral sessions: baseline, conditioning, context retrieval, cue retrieval/extinction, and retention of extinction. Unless otherwise noted, the term “retention session” refers to the retention-of-extinction session throughout this paper. Thirty-second 5 kHz tone pips (250 ms on, 750 ms off, 74 dB) were used as conditioned stimuli (CS), and trains of 16 electrical pulses (4.6–5.1 mA, 2 ms pulse width, presented at 8 Hz) to the eyelids (Johansen et al., 2010) were used as unconditioned stimuli (US). In each behavioral session, rats were allowed to freely explore the behavioral chambers for 4 min, during which no CS or US were presented. In context-retrieval sessions, rats were returned to their home cages immediately after the 4 min free-exploration period. In the baseline, conditioning, cue-retrieval/extinction, and retention sessions, the first CS was presented immediately after the 4 min exploration period. The total numbers of CS presentations were 4, 12, 40, and 8, respectively. Although cue-retrieval and extinction sessions were conducted continuously without interruption, the period beginning with the ninth CS—during which the inter-CS intervals were shorter than in the earlier part of the session—was designated as the extinction session. Inter-CS intervals were uniformly distributed between 180 and 240 s, except for those during the extinction session, where they ranged from 60 to 120 s. Only during the conditioning session, US followed each CS with a trace interval of 700–750 ms. Cue-retrieval and extinction sessions were analyzed separately, unless otherwise stated.

Immediately after the recording sessions, the rats were anesthetized with isoflurane, and microlesions were created by applying a 3 μA DC current for 10 s to the top and bottom recording sites of each shank. Histological reconstruction of probe positions was reported in a previous study (Miyawaki and Mizuseki, 2022).

Automatic spike sorting was performed with Kilosort2 (Pachitariu et al., 2024; http://github.com/MouseLand/Kilosort2), then spikes detected around each shock pulse (0.1 ms prior to onset to 5 ms following the offset of each pulse) were discarded to exclude potential contamination by shock artifacts. The clustering results were manually curated using the phy software (https://github.com/cortex-lab/phy), and we adopted units that met all of the following four criteria, as previously reported (Miyawaki and Mizuseki, 2022): (1) isolation distance (Harris et al., 2001) >15, (2) interspike interval index (Fee et al., 1996) <0.2 or contamination rate <0.05, (3) overall mean firing rates >0.01 Hz, and (4) spike amplitudes >50 μV.

Among well-isolated units, putative excitatory and inhibitory neurons were identified by detecting monosynaptic interactions using the previously described method (Fujisawa et al., 2008) with minor modifications (Miyawaki and Mizuseki, 2022). Briefly, for each pair of cells recorded simultaneously from the same brain region, a spike-timing cross-correlogram (CCG) was computed using 0.1 ms bins and smoothed with a Gaussian kernel (*σ* = 0.5 ms). The same procedure was applied to randomly jittered spike trains of the same cell pair, with jitter values distributed uniformly within ±5 ms, and the maxima and minima of the CCG within the ±5 ms range were detected. This procedure was repeated 1,000 times to construct 99% global bands (the 0.5th and 99.5th percentiles of the CCG minima and maxima, respectively, obtained from the jittered spike trains). Pairs whose actual CCGs exhibited peaks higher than the upper bound or troughs lower than the lower bound of the 99% global bands within the [+1, +4] ms range were considered pairs with excitatory or inhibitory monosynaptic interactions, respectively. Neurons that sent at least one excitatory output but no inhibitory output were classified as putative excitatory cells. Likewise, neurons that sent at least one inhibitory output but no excitatory output were classified as putative inhibitory cells. Nearly all neurons identified as excitatory and inhibitory by the CCG-based method exhibited spike waveforms with a full-width at half-maximum (FWHM) >0.6 ms and <0.5 ms, respectively. Therefore, following previously reported methods (Barthó et al., 2004), cells that could not be classified based on CCGs were categorized according to their spike FWHM (>0.6 ms, excitatory; <0.5 ms, inhibitory), and any remaining cells were marked as non-classified (Miyawaki and Mizuseki, 2022).

### Freeze detection

As described in a previous study (Miyawaki and Mizuseki, 2022), periods of freezing behavior were detected using a Gaussian mixture hidden Markov model with three hidden states. The model was trained using heart rate, 0.5–5 Hz and 5–10 Hz band power of the EOG, nuchal EMG amplitudes, and the logarithm of head acceleration. Among the three hidden states, the one with the lowest mean head acceleration was labeled as the candidate freezing state. Periods in which the model remained in the candidate freezing state for >5 s were classified as freezing periods. Freezing periods separated by gaps <1 s were merged.

### Identification of local ensembles and detection of their activation and inter-regional coactivation

Independent component analysis (ICA)-based ensemble identification, detection of ensemble activation events, and evaluation of inter-regional coactivation of local ensembles were performed using previously deposited MATLAB code (Miyawaki and Mizuseki, 2022). Briefly, ensemble detection was performed in each region of interest for each rat, separately, and all simultaneously recorded units (putative excitatory, inhibitory, and non-classified cells) in a region of interest were included in the detection. Ensemble detection was performed separately for the conditioning, extinction, and retention sessions, and the resultant ensembles were termed conditioning-, extinction-, and retention-ensembles, respectively. The firing rate of individual units was calculated in 20 ms bins during template epochs (i.e., conditioning, extinction, or retention sessions) and *z*-scored within each unit. The number of significant components was defined as the number of eigenvalues of the correlation matrix of the *z*-scored firing rate matrix that exceeded the Marchenko–Pastur threshold (Peyrache et al., 2009; Lopes-dos-Santos et al., 2013; Farooq et al., 2019; Giri et al., 2019; Oliva et al., 2020; Fernández-Ruiz et al., 2021; Miyawaki and Mizuseki, 2022; Zutshi et al., 2022; Harvey et al., 2023). Using this number of significant components, ICA was applied to the *z*-scored firing rate matrix to identify neuronal ensembles (Lopes-dos-Santos et al., 2013; van de Ven et al., 2016; Giri et al., 2019; Oliva et al., 2020; Miyawaki and Mizuseki, 2022; Zutshi et al., 2022; Harvey et al., 2023). The sign of each projection vector was determined such that its component with the largest absolute value was positive. Significantly contributing cells for each ensemble were defined as those with *z*-scored weights >2.0, where *z*-scoring was performed within each projection vector (van de Ven et al., 2016; Farooq et al., 2019; Fernández-Ruiz et al., 2021; Zutshi et al., 2022). The instantaneous activation strength in a matched bin, whose firing rate vector (calculated in 20 ms bins) is denoted as ***M***, was calculated as ***M*^T^*PM***, where ***P*** is the outer product of the projection vector of an ensemble of interest with its diagonal set to zero (Peyrache et al., 2009; Lopes-dos-Santos et al., 2013; Farooq et al., 2019; Giri et al., 2019; Oliva et al., 2020; Fernández-Ruiz et al., 2021; Miyawaki and Mizuseki, 2022; Zutshi et al., 2022; Harvey et al., 2023). Instantaneous activation strength was *z*-scored using the mean and standard deviation (SD) during non-rapid eye movement (NREM) sleep epochs for each ensemble. Ensemble activation events were then detected as peaks in the *z*-scored instantaneous activation strength with amplitudes exceeding 5 *z* (Peyrache et al., 2009; Farooq et al., 2019; Oliva et al., 2020; Miyawaki and Mizuseki, 2022). The variability of the ensemble activation event rates was evaluated using the coefficient of variation (CV), which was calculated by dividing the SD of interevent intervals by the mean interevent intervals.

Ensemble pairs that were coactivated across regions during NREM sleep were identified based on the peak height of CCGs between *z*-scored instantaneous activation strengths of ensembles within a ± 100 ms range (Miyawaki and Mizuseki, 2022). The significance of the peak height was examined in comparison with the null distribution of peak height obtained by chunk (2 s) shuffling, and ensemble pairs whose CCG peaks were larger than the 99.5th percentile of the null distribution were considered coactivated pairs (Miyawaki and Mizuseki, 2022). To assess the variability of coactivation across the rats, we compared the fraction of coactivated ensemble pairs for each rat with the corresponding leave-one-out estimate derived from the pooled data of the remaining animals, using *χ*^2^ tests with Bonferroni correction (Fig. S2*B*). The 95% confidence intervals for the proportions of coactivated pairs were calculated using Wilson’s score method. Ensembles participating in at least one coactivated pair were referred to as coactivated ensembles, and the remaining were referred to as non-coactivated ensembles.

To detect coactivation events of the inter-regionally coactivated ensemble pairs during NREM sleep, we computed the instantaneous coactivation strength, which was defined as the product of the *z*-scored instantaneous activation strengths of the two ensembles, with one shifted by the time lag corresponding to the CCG peak within the ±100 ms range used for the identification of inter-regionally coactivated ensemble pairs described above (Miyawaki and Mizuseki, 2022). Coactivation event timestamps were identified as the peak times of the instantaneous coactivation strength that exceeded 25 *z*^2^.

### Determination of significantly similar ensembles

To compare local ensembles detected across different template periods, we calculated the absolute value of the cosine similarity between their corresponding projection vectors, with cell IDs aligned across periods. The absolute value was used because the sign of ICA projection vectors is arbitrary, making the sign of the cosine similarity meaningless. Because the distribution of cosine similarity among random vectors depends on their dimensionality (Fig. 2*D*), we assessed the significance of absolute cosine similarity separately for each rat and each brain region. Specifically, projection vectors from ensembles identified during the conditioning, extinction, and retention sessions were pooled. Subsequently, 5,000 vector pairs were drawn with replacement from the pool, and their cell IDs were independently shuffled. The 99th percentile of the absolute cosine similarity from these shuffled surrogates was used as the significance threshold. Ensemble pairs from different template epochs whose absolute cosine similarity exceeded this threshold were considered significantly similar.

For visualization purpose (Fig. 2*C*), the order of extinction- and conditioning-ensembles was rearranged to align the components with the largest absolute cosine similarity between their weight vectors along the diagonal of the similarity matrix. First, the global maximum of the similarity matrix was identified. Then, the rows and columns and, thus, the order of the extinction- and conditioning-ensembles were swapped to position the maximum in the top-left corner. The same operation was iteratively applied to the remaining submatrices until the entire matrix was reordered. Retention-ensembles were sorted similarly but with the order of extinction-ensembles fixed and only the columns of the similarity matrix were permuted; for the first extinction-ensemble, the retention-ensemble with the highest similarity (i.e., the maximum value in the first row) was moved to the first column, and then the retention-ensemble with the highest remaining similarity in the second row was placed in the second column, and so on for the subsequent rows. Cells were subsequently reordered based on the weight vectors of the extinction-ensembles. Each cell was first assigned to the ensemble in which it exhibited the largest weight. Subsequently, the cells assigned to the first ensemble were sorted in a descending order of their weights to define their indices. The same procedure was applied to the second ensemble, whose indices followed those of the first ensemble. This procedure was repeated for all extinction-ensembles. These reordering procedures of ensembles and cells enabled consistent visualization of ensemble correspondence and strength of cell contributions; however, they were performed solely for visualization purposes and did not affect the subsequent analyses.

### Detection of fast network oscillations

Fast network oscillations, i.e., hippocampal SWRs, amygdalar HFOs, and prefrontal cRipples, were detected using a previously published MATLAB code (Miyawaki and Mizuseki, 2022). SWRs were detected during both NREM sleep and wakefulness because they frequently occur during quiet wakefulness and NREM epochs (Buzsáki, 2015), whereas HFOs and cRipples were detected exclusively during NREM epochs because they predominantly occur during this state (Ponomarenko et al., 2003; Buzsáki, 2015).

SWRs were identified as the co-occurrence of sharp waves and ripples in hippocampal LFPs. Ripples were detected on each hippocampal channel based on ripple-band power, calculated as the root mean square (RMS) of bandpass filtered (100–250 Hz) LFPs in 13.3 ms windows. Ripple-band power was *z*-scored using the mean and SD during NREM epochs. Ripple candidates were identified as periods with *z*-scored power >1.5. Candidates with a peak power <4 *z* or duration <30 ms were excluded, and those separated by gaps <10 ms were combined. Overlapping candidates detected on channels of the same shank were merged. Sharp waves were detected on the differences in LFP signals between the most superficial and deepest channels on the same shank. The differences were calculated by subtracting the LFP of the deepest channel from that of the most superficial channel, subsequently bandpass filtered (2–40 Hz), and *z*-scored using the mean and SD during NREM. Sharp waves were detected as periods with a *z*-scored difference <–2.5, lasting between 20 and 400 ms. Ripple candidates overlapping with at least one sharp wave trough detected on the same shank were classified as SWR candidates. Finally, SWR candidates detected on different shanks were merged, and only those with a duration of <750 ms were retained as SWRs.

HFOs were detected on each amygdalar shank using the median LFP signal within the shank of interest. High-frequency band power was calculated as the RMS of bandpass filtered (90–180 Hz) LFPs in 20 ms time windows and then *z*-scored using the mean and SD during NREM sleep. Periods with *z*-scored power >2 for at least 30 ms and peak power >4 *z* were considered HFO candidates. Adjacent candidates separated by <20 ms were combined, and overlapping candidates detected on different shanks were merged. Candidates with a duration <750 ms were accepted as HFOs.

For cRipple detection, LFPs from each prelimbic channel were bandpass filtered (90–180 Hz), and power was calculated as the RMS in 20 ms time windows. This power was *z*-scored using the mean and SD during NREM epochs. cRipple candidates were defined as periods with *z*-scored power >3. Candidates with peak power <5 *z* or duration <50 ms were excluded. Adjacent candidates separated by <30 ms were combined and overlapping candidates across channels were merged. Only candidates with a duration <750 ms were classified as cRipples.

### Modulation of extinction-ensemble activation by fast oscillatory events

Histograms of extinction-ensemble activation events aligned to the peaks of HFOs, cRipples, and SWRs were generated for NREM epochs during the pre- and post-extinction home-cage sessions to visualize how these network events modulate ensemble activation. The gain of extinction-ensemble activation events during oscillatory events was calculated as the ratio of the mean occurrence rate of activation events during the oscillations of interest (i.e., HFOs, cRipples, or SWRs) to that during the NREM epochs.

### Experimental design and statistical analysis

**Figure 1.**
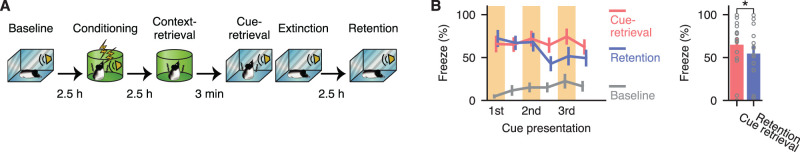
Experimental schedule and animal behavior. ***A***, Time schedule of the behavioral experiment. The behavioral experiment consisted of five sessions: baseline, conditioning, context retrieval, cue retrieval/extinction, and retention. Trains of pip sounds were used as cues and presented during the baseline, conditioning, cue-retrieval/extinction, and retention sessions but not during the context-retrieval session. Electrical shocks to the eyelids were delivered only during the conditioning session. Each session was separated by ∼2.5 h intervals, except for the context- and cue-retrieval/extinction sessions, where the interval was ∼3 min. Cue retrieval and extinction were conducted as one continuous session, whereas inter-cue intervals were shortened during extinction session (see Materials and Methods for details). ***B***, Mean fraction of time in freezing for the first three cue presentations during the baseline, cue-retrieval, and retention sessions (left) and the fraction of time in freezing averaged across the first three cues and the following inter-cue intervals (right). Pale orange and white background colors in the left plot indicate periods of cue presentations and inter-cue intervals, respectively. Gray circles in the right plot represent the fractions of individual rats. Error bars indicate standard errors of the mean. *p < 0.05, Wilcoxon rank-sum test. Details of the statistical tests are provided in Dataset S1.

Throughout the study, we used non-parametric statistical tests because most data were not normally distributed. Metrics for statistical analyses were pooled across cells, ensembles, or ensemble pairs, unless otherwise stated. To examine the significance of the differences in the proportion of time spent in freezing behavior across different behavioral sessions, the Wilcoxon rank-sum test was used (Fig. 1*B*). To test the significance of the differences in the proportion of ensemble categories, the *χ*^2^ test with Bonferroni correction was applied (Fig. 3*A*). Whether the fraction of coactivated ensemble pairs in each rat differed from that in the pooled data of the other rats was tested using the *χ*^2^ test with Bonferroni correction (Fig. S2*B*). Fractions of inhibitory cells among contributing cells of each ensemble (Fig. S4*D*) were compared using the *χ*^2^ test. Correlations between firing rates and their weights in projection vectors were assessed using Spearman’s rank-order correlation coefficients (Figs. S1*E*, S4*A*). To compare ensemble activation rates across different sessions within each ensemble category (Fig. 3*B*) and firing rates across different phases of extinction (Fig. S1*A*,*B*), the Friedman test was used, and if a significant difference was detected (*p* < 0.05), post hoc pairwise comparisons were conducted using the Nemenyi test. Differences in the fractions of coactivated ensemble pairs (Fig. 4*B*) and preserved-ensembles (Fig. 5*A*; Fig. S3*A*) were examined using Fisher’s exact probability test. To examine the significance of differences in ensemble activation rates during fast oscillatory events (Fig. 5*C*; Fig. S3*B*), the Mann–Whitney *U* test with Bonferroni correction was performed. Significance of differences in firing rates between the ensemble contributing and noncontributing cells (Fig. S1*D*) and that between contributing cells of preserved- and attenuated-ensembles (Fig. S4*B*) were tested using the Mann–Whitney *U* test. The Mann–Whitney *U* test was also applied to compare the CV of ensemble activation event intervals between preserved- and attenuated-ensembles (Fig. S4*E*). The increase in ensemble activation events during fast oscillatory events was assessed using the Wilcoxon signed-rank test (Fig. S5*B*). The normality of the weight distribution of projection vectors was evaluated using the one-sample Kolmogorov–Smirnov test (Fig. S1*C*). Distributions of projection vector weights in excitatory and inhibitory cells were compared using the two-sample Kolmogorov–Smirnov test (Fig. S4*C*). When Bonferroni correction yielded adjusted *p* values >1.00, they were truncated and reported as 1.00. Details of the statistical tests are provided in Dataset S1.

### Code accessibility

Data were analyzed using previously published MATLAB code (Miyawaki and Mizuseki, 2022; https://github.com/HiroMiyawaki/Miyawaki2022_NatCommun) and newly developed Python code (https://github.com/HiroMiyawaki/Miyawaki2025), both of which are freely available on GitHub.

## Results

### Assessment of similarity in ensembles detected in different behavioral sessions of the fear-conditioning and extinction experiment

We analyzed previously obtained single-unit activity (232, 479, and 143 units from the BLA, PL5, and vCA1, respectively; see also Table 1 and Table S1) and LFP data from 15 male Long–Evans rats (Miyawaki and Mizuseki, 2022). During the ∼17 h continuous recording period, five behavioral sessions were conducted: baseline, conditioning, context-retrieval, cue-retrieval/extinction, and retention sessions (Fig. 1*A*; see Materials and Methods for details). In the baseline session, the rats were introduced to a novel environment and exposed to trains of novel pip sounds. Following a ∼2.5 h rest/sleep session in the home-cage, the conditioning session was conducted in a different novel environment, where cue sounds were paired 12 times with electrical shocks to the eyelids (Johansen et al., 2010). After another ∼2.5 h home-cage session, the rats were returned to the shock-associated context for the context-retrieval session, during which no cues were presented. Immediately after (∼3 min) the context-retrieval session, the cue-retrieval/extinction session was conducted in the same chamber used for the baseline session, during which the animals were repeatedly exposed to cue sounds without shocks. After an additional ∼2.5 h home-cage session, the rats were reintroduced to the same chamber and exposed to cue sounds to assess whether extinction learning had been retained (retention session). We observed a slight but significant decrease in the proportion of time spent freezing during the retention session compared with that during the cue-retrieval session (*T* = 22.0; *n* = 15; *p* = 0.030, Wilcoxon rank–sum test; Fig. 1*B*), indicating that the animals retained extinction learning at least until the retention session.

**Table 1.** Number of recorded neurons

	Putative excitatory cells	Putative inhibitory cells	Non-classified cells
BLA	209	18	5
PL5	418	50	11
vCA1	92	46	5

The numbers of recorded neurons in each region are shown. See also Table S1.

**Figure 2.**
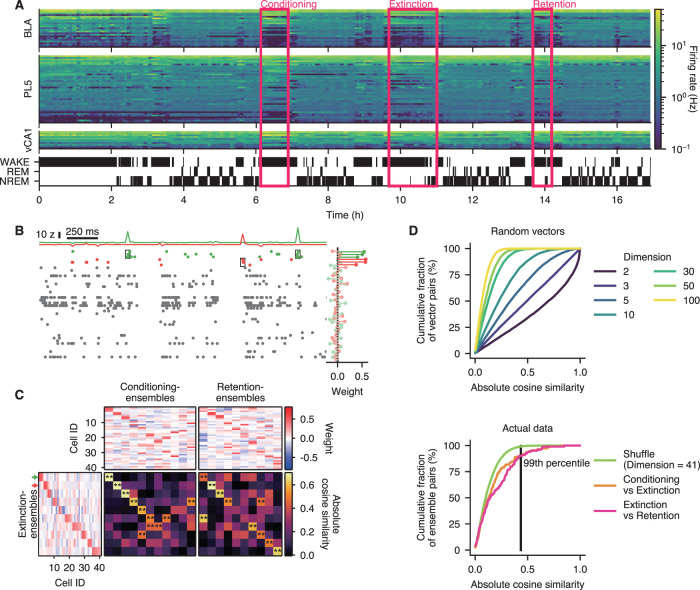
Assessment of ensemble similarity across behavioral sessions. ***A***, Data recorded from an example rat (Rat M in Table S1). Unit activities and LFPs were simultaneously recorded from the PL5, BLA, and vCA1 for ∼17 h. For visualization, mean firing rates were calculated for each unit in 1 min bins, and each row represents each unit. All simultaneously recorded units from the example rat (23, 41, and 11 units for BLA, PL5, and vCA1, respectively) are shown. Conditioning, extinction, and retention sessions were conducted during the recording period (see Materials and Methods for details), and their timings are indicated using magenta rectangles. ***B***, Example raster plots of PL5 units recorded from the example rat (41 units from Rat M) shown in ***A*** and two representative extinction-ensembles. The weights of each unit and the instantaneous activation strength of ensembles are shown on the right and at the top, respectively. Horizontal and vertical scale bars indicate time and instantaneous activation strength of ensembles, respectively. In the raster plot, units with significant contributions to each ensemble (*z*-scored weight >2.0; see Materials and Methods for details) are displayed as colored dots, whereas the remaining units are shown as gray dots. Spikes of significantly contributing units within ensemble activation events are indicated using black rectangles. In the right plot, the weight of significantly contributing units and the remaining units are shown in vivid and pale colors, respectively. Small vertical offsets were added to the instantaneous activation strengths (top) and weights (right) to improve visibility. ***C***, Ensemble similarity analysis. As an example, analysis for the PL5 data of the example rat (Rat M) shown in (***A***) is presented. Absolute cosine similarities between extinction- and conditioning-ensembles are shown on the left, and those between extinction- and retention-ensembles are shown on the right. Cell weights for extinction-, conditioning-, and retention-ensembles are presented on the bottom left, top left, and top right, respectively. Ensembles were sorted by similarity, and cell IDs were assigned based on weights of extinction-ensembles (see Materials and Methods for details). The same cell IDs were also used for conditioning- and retention-ensembles. Colored arrows indicate ensembles shown in ***B***. **p < 0.01, shuffling test for absolute cosine similarity. ***D***, Cosine similarity is influenced by data dimensionality. Top, Cumulative fractions of the absolute cosine similarity for 10,000 randomly generated vector pairs, with each element in the vectors drawn independently from a uniform [0, 1] distribution, are shown for 2-, 3-, 5-, 10-, 30-, 50-, and 100-dimensional vectors. Note that cosine similarity values tend to cluster more closely around zero in higher-dimensional spaces. Bottom, Actual distribution of cosine similarity for the PL5 ensembles of the example rat (Rat M, shown in ***C***) and its shuffled surrogates (with 41 cells). The vertical line indicates the 99th percentile of the shuffled surrogate distribution, and pairs above this line are regarded as significantly similar.

While this study primarily focused on extinction sessions, we also analyzed the conditioning, retention, and interleaving home-cage sessions (Figs. 1, 2; Fig. S1*A*,*B*). Excitatory cells exhibited only minor changes in their firing rates. The firing rates of BLA excitatory cells in the retention session were higher than those in the conditioning session but lower than those in the extinction session (*q* = 8.02 and 5.05; *n* = 209; *p* = 4.19 × 10^−8^ and 0.001 for conditioning vs retention and extinction vs retention, respectively, post hoc Nemenyi test following Friedman test; Fig. S1*A*). However, the firing rates of vCA1 excitatory cells during NREM sleep were higher in the pre-conditioning than in the pre-extinction home-cage sessions (*q* = 4.85; *n* = 92; *p* = 0.003, post hoc Nemenyi test following Friedman test; Fig. S1*A*). Inhibitory cells also showed subtle changes in firing rates. The firing rates of BLA inhibitory cells were higher in the conditioning session than in other behavioral sessions (*q* = 5.42 and 4.48; *n* = 18; *p* = 3.72 × 10^−4^ and 0.004 for conditioning vs extinction and conditioning vs retention, respectively, post hoc Nemenyi test following Friedman test; Fig. S1*B*). These results indicate that extinction sessions induced only minor modifications in firing rates.

Relatively small groups of coactive neurons, known as cell ensembles, cell assemblies, or engram cells, are believed to represent specific information through their coordinated activity (Harris, 2005; Tonegawa et al., 2018). We identified local cell ensembles using an ICA-based method (Fig. 2*B*), as described previously (Peyrache et al., 2009; Lopes-dos-Santos et al., 2013; van de Ven et al., 2016; Giri et al., 2019; Oliva et al., 2020; Fernández-Ruiz et al., 2021; Miyawaki and Mizuseki, 2022; Harvey et al., 2023). By this method, the number of ensembles for each brain region was estimated based on the eigenvalues of the neuronal firing correlation matrix. Then, using ICA, the same number of components was extracted from the *z*-scored firing rate matrix of neurons, and each component is referred to as a local cell ensemble (see Materials and Methods for details). We detected cell ensembles separately during the conditioning, extinction, and retention sessions. Hereafter, local cell ensembles detected during the conditioning, extinction, and retention sessions are referred to as conditioning-, extinction-, and retention-ensembles, respectively. Within each region, the numbers of conditioning-, extinction-, and retention-ensembles were comparable (Table 2; Table S1). For each ensemble, significantly contributing cells were determined based on their weights of the projection vector (Fig. 2*B*; see Materials and Methods for details). The distribution of weights was significantly skewed from the normal distribution (*D* = 0.16, 0.14, and 0.12; *n* = 2,066, 5,898, and 724; *p* = 1.75 × 10^−46^; 9.17 × 10^−97^; and 1.94 × 10^−9^ for BLA, PL5, and vCA1, respectively, one-sample Kolmogorov–Smirnov test; Fig. S1*C*), reflecting that only a small fraction of the cells predominantly contributed to each ensemble (Tonegawa et al., 2015; Josselyn and Tonegawa, 2020). Mean firing rates did not differ significantly between contributing and remaining cells (*p* > 0.21, Mann–Whitney *U* test; Fig. S1*D*), and no significant correlation was found between firing rates and projection vector weights, except with vCA1 inhibitory cells (*ρ* = 0.13; *n* = 239; *p* = 0.045, the significance test for Spearman’s correlation coefficient; Fig. S1*E*), suggesting that neuronal firing rate alone did not account for ensemble composition.

**Table 2.** Number of detected ensembles

	Conditioning-ensembles	Extinction-ensembles	Retention-ensembles
BLA	60	64	58
PL5	93	95	84
vCA1	38	33	35

The numbers of ensembles detected in each region during the conditioning, extinction, and retention sessions are shown (see also Table S1).

First, we tested whether extinction-ensembles were maintained across behavioral sessions by calculating the absolute cosine similarity between the weight vectors of ensembles, using the weights of all simultaneously recorded cells within the region where the ensembles were detected (Fig. 2*C*). This analysis revealed that some extinction-ensembles had one or a few highly similar partners among the conditioning- and/or retention-ensembles, suggesting that certain ensembles were maintained across behavioral sessions. As the distribution of absolute cosine similarity depends on vector dimensionality (Fig. 2*D*), we generated a null distribution of absolute cosine similarity for each brain region and each rat individually using a shuffling method (see Materials and Methods for details). If the absolute cosine similarity between two ensembles exceeded the 99th percentile of the null distribution, the ensembles were considered significantly similar (Fig. 2*C,D*). These analyses revealed that while some ensembles were maintained across behavioral sessions, others either emerged or disappeared—presumably due to experience-dependent modifications of local neuronal circuits.

### Ensemble compositions in the PL5 were more stable than those in the vCA1 and BLA

**Figure 3.**
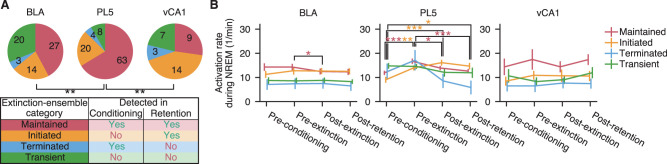
PL5 ensemble compositions were more stable than those in other brain regions, whereas ensemble activities varied across home-cage sessions. ***A***, Classification and fractions of ensemble categories. Extinction-ensembles were classified into four categories: maintained (red), initiated (orange), transient (blue), or terminated (green), based on whether at least one significantly similar ensemble was detected during conditioning and/or retention sessions (bottom). The fractions of these ensemble categories are summarized for each brain region (top), with the number of ensembles superimposed. **p < 0.01, *χ*^2^ test with Bonferroni correction. ***B***, Mean activation event rates of ensembles during NREM sleep. Event rates are shown for concatenated NREM epochs during pre-conditioning, pre-extinction, post-extinction, and post-retention home-cage sessions. Small horizontal jitters have been added to improve the visibility of each line. Colors of lines and asterisks correspond to ensemble categories. Ensembles were pooled across all examined rats, and their mean and standard error of the mean are presented. ***p < 0.001; **p < 0.01; *p < 0.05; Nemenyi test. Details of the statistical tests are provided in Dataset S1.

As described above, some extinction-ensembles were maintained across behavioral sessions, while others either emerged or disappeared. To further characterize ensemble reorganization in each brain region, we classified extinction-ensembles into the following four categories based on whether the ensemble of interest had similar partners in the conditioning- and/or retention-ensembles (Fig. 3*A*): (1) maintained, extinction-ensembles with similar partners in both conditioning- and retention-ensembles; (2) initiated, extinction-ensembles with similar partners in retention-ensembles but not in conditioning-ensembles; (3) terminated, extinction-ensembles with similar partners in conditioning-ensembles but not in retention-ensembles; and (4) transient, extinction-ensembles with no similar partners in either conditioning- or retention-ensembles. All four categories were observed in each recorded brain region; however, the proportions of these categories differed significantly across regions (*χ*^2^_(3)_ = 15.28 and 15.33; *n* = [64, 95] and [95, 33]; *p* = 0.005 and 0.005 for BLA vs PL5 and PL5 vs vCA1, respectively, *χ*^2^ test with Bonferroni correction; Fig. 3*A*), presumably reflecting regional differences in the flexibility of local neuronal circuits. Notably, PL5 exhibited a higher proportion of maintained-ensembles, whereas vCA1 showed a higher proportion of initiated-ensembles. These findings suggest that hippocampal circuits are more prone to rapid changes, while prelimbic cortical circuits undergo more gradual reorganization.

Neuronal circuits are reorganized during sleep to support memory consolidation (Stickgold and Walker, 2007; Diekelmann and Born, 2010); therefore, we hypothesized that extinction-ensembles preserved until the retention session would exhibit enhanced activity during post-extinction sleep. To test this, we identified individual activation events of each extinction-ensemble and calculated their mean activation event rates during NREM sleep epochs (Fig. 3*B*). Notably, in the PL5, both maintained- and initiated-ensembles showed increased activity during pre-extinction sleep, with initiated-ensembles maintaining this elevated activity through at least the post-retention sleep epochs (*q* = 7.51, 4.00, and 6.64; *n* = 63; *p* = 6.43 × 10^−7^, 0.024, and 1.60 × 10^−5^ for pre-conditioning vs pre-extinction, pre-extinction vs post-extinction, and pre-extinction vs post-retention, respectively, among maintained-ensembles; and *q* = 4.50, 6.41, and 4.33; *n* = 20; *p* = 0.008; 3.47 × 10^−5^, and 0.012 for pre-conditioning vs pre-extinction, pre-conditioning vs post-extinction, and pre-conditioning vs post-retention, respectively, among initiated-ensembles, post hoc Nemenyi test following Friedman test). In contrast, no experience-dependent increase in ensemble activity was observed in the BLA or vCA1. In fact, maintained-ensembles in the BLA exhibited decreased activation during post-extinction sleep compared with that during pre-extinction sleep (*q* = 4.02; *n* = 27; *p* = 0.023; post hoc Nemenyi test following Friedman test). These findings suggest that ensemble activation during sleep alone is not sufficient to determine whether ensemble compositions are preserved into subsequent wakefulness.

### Extinction did not enhance inter-regional coactivation but reorganized inter-regional coactivation pairs

**Figure 4.**
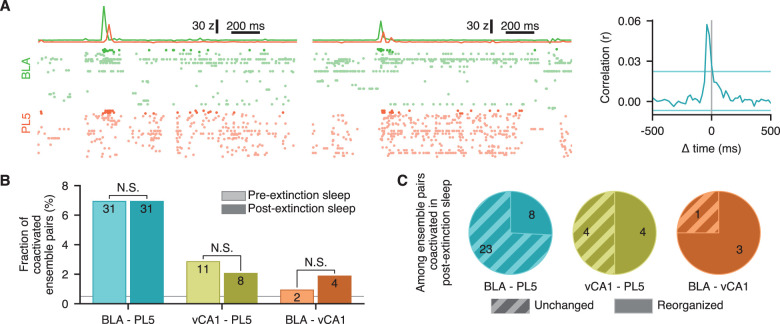
Extinction did not enhance inter-regional coactivation but reorganized coactivation pairs. ***A***, Example of an inter-regional extinction-ensemble pair coactivated during post-extinction sleep from a representative example rat (Rat H in Table S1). The left two panels show raster plots of BLA and PL5 spikes around coactivation events with the instantaneous activation strengths of the ensembles superimposed on top. Small vertical offsets were added to the instantaneous activation strength to improve visibility. Spikes from significantly contributing units (*z*-scored weight >2.0; see Materials and Methods for details) are shown in vivid colors, while spikes from the remaining units are shown in pale colors. Horizontal and vertical scale bars indicate time and instantaneous activation strength of ensembles, respectively. The rightmost plot illustrates the CCG of the example ensembles’ instantaneous activation strengths during NREM sleep in the post-extinction home-cage session. Horizontal bars represent the 0.5th and 99.5th percentiles of CCG peaks and troughs obtained from shuffled surrogates. Ensemble pairs with CCG peaks higher than the 99.5th percentiles were regarded as coactivated pairs. ***B***, Proportion of extinction-ensemble pairs that were significantly coactivated during NREM sleep in pre- (light colors) and post-extinction (dark colors) home-cage sessions, relative to all ensemble pairs. The numbers of coactivated ensemble pairs are superimposed. The horizontal gray line indicates the chance level (0.5%). Note that there was no significant increase in inter-regional coactivations for ensembles detected during extinction session. N.S., not significant (*p* > 0.05), Fisher's exact probability test. ***C***, Reorganization of ensemble coactivation pairs. The fraction of ensemble pairs that were coactivated during pre-extinction NREM epochs (unchanged pairs) and those that were not (reorganized pairs) among the ensemble pairs that showed significant coactivation during the post-extinction NREM sleep. The numbers of ensemble pairs are superimposed. Details of the statistical tests are provided in Dataset S1.

We previously reported that inter-regional coactivation of conditioning-ensembles is enhanced following fear conditioning (Miyawaki and Mizuseki, 2022). Therefore, we investigated whether extinction-ensembles exhibited increased inter-regional coactivation during post-extinction sleep compared with pre-extinction sleep. Coactivated ensemble pairs were determined based on the CCG peak height of instantaneous activation strengths (Fig. 4*A*; Fig. S2; see Materials and Methods for details). No significant variability was observed in the fraction of coactivated ensemble pairs across rats (Fig. S2*B*). We found that a small fraction of extinction-ensembles were inter-regionally coactivated during both pre- and post-extinction home-cage sessions (Fig. 4*A,B*); however, no significant changes were observed in the proportion of coactivated ensemble pairs (*p* > 0.64 for all examined brain regions, Fisher's exact probability test; Fig. 4*B*). Notably, results in BLA-vCA1 should be interpreted with caution, as the number of coactivated pairs was small, although their proportion was higher than expected by chance. The lack of change in the proportion of coactivating pairs does not necessarily mean that the same coactivation occurs in both epochs; the coactivation pattern may have changed. Therefore, we examined whether the coactivation partners of ensembles remained consistent between pre- and post-extinction sleep. Remarkably, a substantial fraction of coactivated ensembles changed partners (Fig. 4*C*). These findings suggest that, although the extinction session did not enhance inter-regional coactivation, it may have altered the fine structure of inter-regional circuit connectivity.

### Extinction-ensembles contributing to inter-regional coactivation tended to be preserved until the retention session

**Figure 5.**
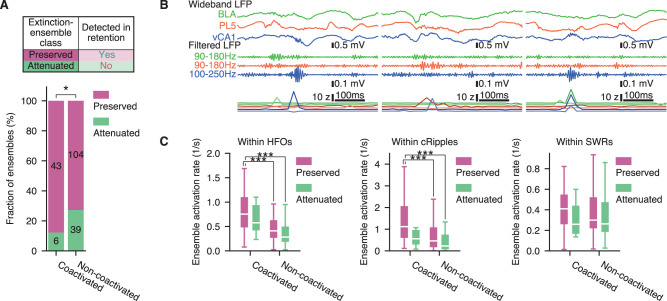
Extinction-ensembles contributing to inter-regional coactivation tended to be preserved until the retention session. ***A***, Fractions of preserved and attenuated extinction-ensembles. A preserved-ensemble is one with at least one significantly similar ensemble detected in the retention session, while an attenuated-ensemble is one with no significantly similar ensemble detected in the retention session. Ensembles were summed across regions, and their fractions were summarized by ensemble type: coactivated [ensembles with at least one coactivation partner in other brain region(s) during post-extinction NREM sleep] and non-coactivated (the remaining ensembles). *p < 0.05, Fisher's exact probability test. See also Figure S3. ***B***, Examples of high-frequency oscillatory events and ensemble activation during the post-extinction home-cage session. Simultaneously recorded wideband and bandpass-filtered traces are shown at the top and middle, respectively. The bottom traces display the instantaneous activation strengths of the two representative ensembles from each region. Small vertical offsets were added on the instantaneous activation strength traces to improve visibility. Line colors indicate the corresponding brain regions. Horizontal scale bars indicate time. Vertical scale bars indicate from top to bottom, the amplitude of wideband LFP, amplitude of filtered LFP, and ensemble activation strength. ***C***, Ensemble activation rates during amygdalar HFOs (left), prelimbic cRipples (middle), and hippocampal SWRs (right) in the post-extinction NREM sleep. ***p < 0.001, Mann–Whitney *U* test with Bonferroni correction. See also Figure S3. Details of the statistical tests are provided in Dataset S1.

As described above, the activation rate alone did not account for whether ensembles were preserved (Fig. 3). Additionally, while the extinction session changed coactivation partners, it did not enhance overall coactivation (Fig. 4). Based on these observations, we hypothesized that not merely ensemble activation but rather coactivation—particularly with ensembles in other brain regions—may be critical for ensemble preservation. To test this hypothesis, we categorized extinction-ensembles based on whether they were preserved until the retention session (Fig. 5*A*). Hereafter, extinction-ensembles that had significantly similar retention-ensembles and the remaining are referred to as preserved- and attenuated-ensembles, respectively. Preserved-ensembles correspond to the combination of maintained- and initiated-ensembles defined above (Fig. 3*A*), whereas attenuated-ensembles correspond to the terminated- and transient-categories. Then, we compared the proportion of preserved-ensembles between those with at least one coactivation partner in another brain region during the post-extinction home-cage session (coactivated ensembles) and those with no detectable partner (non-coactivated ensembles; Fig. 5*A*). As expected, the fraction of preserved-ensembles was higher among coactivated ensembles than among non-coactivated ones (odds ratio = 0.372; *n* = [49, 143]; *p* = 0.03; Fisher's exact probability test). This trend was consistent across all brain regions examined (Fig. S3*A*). These findings suggest that ensemble stability is associated with participation in inter-regional coactivation.

In contrast, the mean firing rates and projection vector weights of the neurons were not correlated for preserved- and attenuated-ensembles (*p* > 0.086 for all examined regions, significance test for Spearman's rank-order correlations; Fig. S4*A*). Additionally, the firing rates of significantly contributing cells were comparable between preserved- and attenuated-ensembles (*p* > 0.06 for all examined regions, Mann–Whitney *U* test; Fig. S4*B*). These results indicate that firing rates alone did not directly determine whether an ensemble was preserved. Among preserved-ensembles but not attenuated-ones, inhibitory cells tended to have larger weights than excitatory cells (*U* = 0.15, 0.10, and 0.19; *n* = [1,524, 120], [4,496, 557], and [351, 160]; *p* = 0.009; 1.30 × 10^−4^ and 8.64 × 10^−4^ for preserved-ensembles in BLA, PL5, and vCA1, respectively; *p* > 0.347 for attenuated-ensembles, Mann–Whitney *U* test; Fig. S4*C*). In contrast, the fraction of inhibitory cells among significantly contributing cells did not differ significantly between preserved- and attenuated-ensembles (*p* > 0.053 for all examined brain regions, Mann–Whitney *U* test; Fig. S4*D*). These results suggest that inhibitory cells contributing to an ensemble play an important role in preserving the ensemble; however, this effect is unlikely to be solely due to the greater number of inhibitory cells, leading to more stable ensembles. Taken together, these findings indicate that the firing properties of contributing cells alone cannot fully account for the ensemble preservation. Additional factors, such as inter-regional coactivation, likely play a critical role in stabilizing local ensembles.

Inter-regional coactivation predominantly occurred during high-frequency network oscillations, such as hippocampal SWRs, amygdalar HFOs, and prelimbic cRipples (Fig. 5*B*; Fig. S5; Miyawaki and Mizuseki, 2022). Based on this observation, we hypothesized that ensemble reactivations during these network oscillatory events play a crucial role in stabilizing local ensembles. Consistent with this hypothesis, preserved-ensembles among coactivated ensembles tended to be more frequently reactivated during the post-extinction home-cage session than preserved- or attenuated-ensembles among non-coactivated ensembles (Fig. 5*C*). This trend was particularly evident during HFOs (*U* = 3,264 and 1,289; *n* = [43, 104] and [43, 39]; *p* = 7.28 × 10^−5^ and 1.76 × 10^−4^ for coactivated-preserved vs non-coactivated-preserved and coactivated-preserved vs non-coactivated-attenuated, respectively, Mann–Whitney *U* test with Bonferroni correction) and cRipples (*U* = 3,268 and 1,401; *n* = [43, 104] and [43, 39]; *p* = 6.73 × 10^−5^ and 1.08 × 10^−6^ for coactivated-preserved vs non-coactivated-preserved and coactivated-preserved vs non-coactivated-attenuated, respectively, Mann–Whitney *U* test with Bonferroni correction), although it was less pronounced during SWRs (*p* > 0.367 for all ensemble category pairs, Mann–Whitney *U* test with Bonferroni correction). Furthermore, in the vCA1, preserved-ensembles among coactivated ensembles showed greater reactivation than attenuated-ensembles among non-coactivated ensembles during HFOs and cRipples (*U* = 63.0 and 60.0; *n* = [7, 10]; *p* = 0.014 and 0.041 for coactivated-preserved vs non-coactivated-attenuated during HFOs and cRipples, respectively, Mann–Whitney *U* test with Bonferroni correction; Fig. S3*B*) but not during SWRs (*p* > 0.360 for all examined ensemble category pairs, Mann–Whitney *U* test with Bonferroni correction; Fig. S3*B*). Given that vCA1 neurons send dense efferent projections to the PL5, while reciprocal projections are sparse or absent (Jay and Witter, 1991; Tovote et al., 2015), these findings suggest that entrainment of an ensemble to oscillatory events in downstream regions—rather than those occurring in the region where the ensemble was detected—may exert a greater influence on ensemble stability. Furthermore, in BLA and vCA1, the activation rate of preserved-ensembles fluctuated more on shorter time scales (*U* = 614.0 and 173.0; *n* = [41, 23] and [23, 10]; *p* = 0.047 and 0.024 for BLA and vCA1, respectively, Mann–Whitney *U* test; Fig. S4*E*), indicating that clustering of activation events within fast network oscillation may play an important role in stabilizing ensembles. Taken together, these findings suggest that inter-regional coactivation during fast network oscillations serves as a key mechanism for stabilizing local neuronal ensembles, thereby linking network-level communication dynamics to the persistence of ensemble activity.

## Discussion

In this study, we investigated how local neuronal ensembles change across multiple brain regions and found that ensemble compositions in the PL5 are more stable than those in the BLA or vCA1 (Fig. 3). Moreover, ensembles that were coactivated with those in other brain regions were more likely to be preserved, at least until the retention session (Fig. 5). These findings suggest a potential role of inter-regional coactivation in stabilizing local ensembles. The activity of local ensembles is thought to convey various types of information to downstream regions (Buzsáki, 2010), and inter-regional coactivation likely reflects the transfer of such information between brain regions. This implies that the activity of local ensembles involved in inter-regional coactivation may carry more meaningful information for downstream regions than that of non-coactivated ensembles. Therefore, our findings suggest that local ensembles contributing to inter-regional coactivation—thereby supporting inter-regional information transfer—are preferentially preserved, possibly due to their functional importance within the broader neural network.

We found that inter-regionally coactivated ensemble pairs were altered following the extinction session (Fig. 4*C*), presumably reflecting modifications in inter-regional synaptic connections. Indeed, the strengthening of inter-regional synaptic connections is crucial for memory retrieval (Nabavi et al., 2014; Abdou et al., 2018). However, we did not observe an overall increase in inter-regional coactivity; rather, we noted changes in coactivation partners (Fig. 4). These findings suggest that experience-related changes in inter-regional networks may involve both the potentiation and depression of synaptic connections. Nevertheless, our dataset was not suitable for directly examining changes in inter-regional synaptic connections. Thus, further studies are warranted to explore this possibility.

Although cortical networks are known to change over time (Holtmaat and Svoboda, 2009), our analyses revealed that ensemble compositions in the PL5 were more stable than those in the BLA and vCA1 (Fig. 3), suggesting that networks in the PL5 undergo slower changes than those in other brain regions. This finding aligns with previous evidence showing that hippocampal ensembles mature shortly after an experience, whereas the maturation of prefrontal ensembles requires more time (Kitamura et al., 2017) and that synaptic changes in the prefrontal cortex occur one day after the experience (Goto et al., 2021). Moreover, maturation of prefrontal ensembles depends on hippocampal activity (Kitamura et al., 2017). Local reactivation of ensembles may contribute to this gradual maturation process through inter-regional coactivation, because local reactivations during fast network oscillations are known to be prolonged following novel experiences (Giri et al., 2019). Consistently, we found that preserved-ensembles with at least one coactivation partner in other brain regions tended to show increased activation during fast network oscillations (Fig. 5). Taken together, these findings suggest that preserved-ensembles coactivated with other brain regions play a critical role in supporting ensemble maturation in slowly changing regions, such as the prefrontal cortex, which is essential for systems memory consolidation.

Our 17 h continuous recording represents an extended period for electrophysiological measurements; however, the dynamics of extinction-related ensembles captured in this study are limited compared with the entire memory process. The reduction in freezing behavior during the retention session, which was relatively subtle (Fig. 1*B*) compared with what is typically observed in standard protocols, may be attributed to the compressed experimental schedule (Fig. 1*A*). Our data may reflect only the initial phase of behavioral change, while the full maturation of extinction may require a longer period. This weak behavioral effect makes it challenging to analyze the relationship between ensemble dynamics and behavior or to determine whether the observed changes in ensembles are attributed to the passing of time or are specifically triggered by the experience, which constitutes a limitation of the present study. Furthermore, although the infralimbic cortex is involved in extinction learning (Milad and Quirk, 2002; Tronson et al., 2012), this study analyzed activity in the prelimbic cortex, which rather promotes maintenance of fear memory through cooperation with the vCA1/BLA (Sotres-Bayon et al., 2012; Tronson et al., 2012). This suggests that the present results may be more related to the spontaneous recovery of fear rather than extinction itself. Since spontaneous recovery typically occurs over days or weeks following extinction, longer-term recordings are necessary to clarify this issue. Extending the recording duration using recently developed techniques (Steinmetz et al., 2021) offers a promising future direction for gaining a deeper understanding of extinction-ensemble dynamics. Estrous-cycle stage modulates inter-regional functional connectivity (Hernandez-Gonzalez et al., 2016; Schoepfer et al., 2020) and influences fear-extinction performance (Milad et al., 2009; Zeidan et al., 2011). Thus, our results suggest that the estrous-cycle–dependent network states contribute to variability in extinction-ensemble stability, resulting in a degree of fear extinction that depends on the estrous cycle. However, because the present study only included males, inference regarding the estrous cycle necessitates direct testing.

In conclusion, our study suggests that inter-regional coactivation and ensemble preservation are related. Considering that memory-encoding neurons retrogradely enhance the likelihood of incorporating inter-regionally connected upstream neurons into memory-related ensembles (Lavi et al., 2023), our results indicate that inter-regional coactivation plays an important role in stabilizing local ensembles. However, it remains unclear whether ensembles involved in inter-regional coactivation are stabilized as a result of this interaction or whether inherently stable ensembles are more likely to be coactivated than vulnerable ones. To clarify this, further studies involving the perturbation and/or enhancement of inter-regional coactivation are warranted.
